# An examination of the prospective association between physical activity and academic achievement in youth at the population level

**DOI:** 10.1371/journal.pone.0253142

**Published:** 2021-06-11

**Authors:** Mia Papasideris, Scott T. Leatherdale, Kate Battista, Peter A. Hall

**Affiliations:** School of Public Health and Health Systems, University of Waterloo, Waterloo, Ontario, Canada; Linneaus University, SWEDEN

## Abstract

Exercise has significant benefits for brain health and this may have downstream learning benefits for youth. However existing studies looking at links between physical activity and academic achievement are limited by relatively small sample sizes and/or cross-sectional designs. The objective of this study is to determine the direction and magnitude of the association between physical activity and academic achievement in a large prospective sample of adolescents. Linear mixed models with random intercepts and multinomial ordinal generalized estimating equations were employed to analyze the prospective relationship between measures of physical activity and academic achievement from the COMPASS study (*N* = 9,898 linked participant data cases from year 2 (2013–2014) to year 4 (2015–2016)). The linear relationships between minutes of moderate to vigorous physical activity and academic achievement (English: *r* = -.047, *p* < .000; Math: *r* = -.026, *p* = .008) as well as meeting the national physical activity guidelines and academic achievement (English: *est* = -.052, *p* = .004; Math: *est* = -.052, *p* = .028) were negative and trivial in magnitude. Organized sport participation showed slight positive associations with academic achievement indices, but these were also of trivial magnitude. In conclusion, the relationship between physical activity and academic achievement was effectively null in this population sample. Advocacy for physical activity programming for adolescent populations may best be undertaken with reference to lack of net academic achievement cost, rather than presence of benefit, or simply with reference to the many other physical and mental health benefits for youth.

## Introduction

Regular physical activity has physical and mental health benefits, including enhanced cardiorespiratory fitness, a decreased risk of type 2 diabetes, reduced mortality, as well as improved mood and reduced depressive symptoms [1–5]. Regular exercise can also improve the functional and cognitive capacity of older adults [6, 7]. The latter effects appear especially salient for brain regions supporting cognitive control and memory [7–11]. These brain health benefits of exercise are present throughout the lifespan and may be especially important for adolescents whom rely on such functions in the academic sphere [10, 12–14]. Academic success is important for career attainment, career adaptability and income potential [15, 16] and so it is advantageous to understand factors that contribute to achievement in adolescence. Despite the evidence supporting the association between exercise and enhanced brain function [8, 10], it is not clear to what extent such enhancements translate into improved academic achievement.

Reductions in physical activity programming in school curriculums in North America decades ago was sometimes rationalized based on an assumption that physical activity programming competes for time with academic subjects [17, 18]. Policy changes aimed at enhancing academic achievement have historically led to school administration reducing time allotments for physical education [17, 19]. Effectively, this perspective posited a negative effect of physical activity on academic achievement based on time competition between the two. The “brain-benefit” and “time-competition” hypotheses pertaining to physical activity and academic achievement both posit a significant relationship, but in opposite directions. Understanding the direction and magnitude of this relationship is potentially important from the perspective of policy.

Recent studies have supported the hypothesis that physical activity supports brain health. In a sample of adolescents, accelerometer-assessed physical activity predicted improved interference task achievement, in addition to enhanced task-related adaptive response in the right dorsolateral prefrontal cortex when indexed by functional near infrared spectroscopy [20]. However, no predictive effect was found in terms of physical activity and self-reported Math grades, and a slight negative but non-significant association was present between active minutes and English grades [20]. These findings suggest that physical activity may have some cognitive benefit, but that the brain benefits of physical activity may not fully translate into improved achievement, potentially due to the time competition between academics and physical activity programs.

Systematic and meta-analytic reviews of studies employing physical activity interventions have shown variable results ranging from null to small positive effects of physical activity interventions (both acute and long-term) on academic achievement outcomes [19, 21–25]. However, among the studies reviewed there is a high degree of variability in the quality of the study designs, a limited number of studies with sufficient power, as well as a large degree of heterogeneity in the intervention components and academic outcomes assessed [19, 22, 23]. More problematic is the inability to achieve blinding (single or double) in such trials. For these reasons, longitudinal study designs may have something important to add to the evidence base, particularly when they are relatively large in size.

The results of longitudinal studies examining the relationship between physical activity and academic achievement in adolescence have also been variable. Some studies utilizing self-reported measures of activity and academic achievement have found small but positive associations between the two variables [26, 27], while others found no evidence of a relationship [28, 29]. Similarly, among investigations utilizing objective measures of physical activity and/or academic performance, some have found small to moderate associations [26, 30–32], and others found null associations [33]. Longitudinal studies investigating the impact of sport participation on self-reported academic performance have demonstrated positive relationships [34, 35]; however, it is unclear whether physical activity benefits academic achievement, or whether other factors associated with sport participation (e.g., enhanced social support, self-efficacy) impact achievement [36, 37], or whether such findings are affected by academic thresholds for sport participation. The sample sizes have been limited in some cases and the absolute effect sizes have been relatively small in all cases, however.

The current study endeavours to address the limitations of the existing literature through the use a large prospective population-based dataset to evaluate the “time-competition” and “brain-benefit” hypotheses, as well as the possibility of a null effect. We examine the association separately for two continuous indicators of academic achievement (English and Math grades) and three different self-reported parameters of physical activity (minutes of moderate-to-vigorous physical activity, percent achieving national activity guidelines, and organized sport participation). The secondary aim of this investigation is to examine the direction of this relationship, if a significant association is present. In keeping with prior smaller scale studies, we hypothesized that the prospective association between physical activity and academic achievement would be positive in direction and small in magnitude. The large sample size and longitudinal nature of COMPASS study provides statistical power and a more definitive representation of the direction and magnitude of association.

## Materials and methods

### Design

The COMPASS study is an ongoing prospective cohort study (2012–2021) that collects annual hierarchical longitudinal data from a convenience sample grade 9 to 12 secondary students (aged 13–18) in Ontario and Alberta, Quebec and British Columbia, Canada. This study utilizes a quasi-experimental design with replenishment to evaluate multiple health behaviours of high school students, as well as school programs, policies, and/or built environment characteristics [38]. COMPASS employs an in-class whole-school sampling data collection method that is designed to facilitate data-collection across multiple schools [38]. In year 2, 89 schools participated (79 Ontarian schools, 9 Albertan schools), and in year four, 81 schools (72 Ontarian schools, 9 Albertan schools) participated (data from Quebec and British Columbia were not available in these earlier waves).

This study uses data from years 2 (2013–14) and 4 (2015–16) of the COMPASS study where a total of 89 schools were sampled; 79 in Ontario and 10 in Alberta. Details of the COMPASS study, including sampling and data collection are available in print [38] and online (www.compass.uwaterloo.ca). An earlier investigation examining guidelines adherence and lifestyle behaviors in the COMPASS data 2015/16 and 2016/17 waves was previously published [27]; however, this current analysis includes the data from the 2013/14 and 2015/16 waves and uses more detailed measures of physical activity components, including MVPA and sport participation, none of which were used in the previously published analysis. The COMPASS study was approved by the Human Research Ethics Board at the University of Waterloo (ORE #30118).

### Participants

In year 2 (2013–2014) and year 4 (2015–2016) of the COMPASS study, participants were recruited using active-information passive-consent permission protocols. The parents or guardians were mailed an information letter and then were asked to either call or email the COMPASS recruitment leader in the event that they did not want their child to participate, and participants could refuse to participate at any time. In year 2, 45,298 high school students (participation rate 80.2%) participated, while in year 4, 40,436 students (participation rate 79.9%) participated. The majority of missing respondents were from absenteeism or scheduled spares at the time of the data collection.

Participants were linked anonymously over time using a self-generated identification code. Due to the rolling sampling design, only participants who are enrolled in school at both time points are eligible to be anonymously linked for the longitudinal sample. At year 2, 23,932 participants total were eligible to be linked to year 4. From this sample, 11,220 participants were successfully linked across years 2 and 4. Among this group, participants with missing data on any of the measures included in the analysis were excluded (*N* = 1322), resulting in a final longitudinal sample of 9,898 participants. full description of expected linkage rates and reasons for unsuccessful links is described elsewhere [39].

### Data collection tool

The COMPASS student questionnaire (C_q_), is an anonymous, self-administered questionnaire and was used to collect student level data on a number of health-related outcomes such as obesity, sedentary behaviour and substance use, as well as correlates on behaviours and demographic attributes [40]. C_q_ data was collected as large whole-school samples during class time and was therefore designed to be completed in one 30 to 40-minute class period [40]. Survey items were chosen in order to meet both science- based and practice-based concerns. The COMPASS questionnaire can be found elsewhere [41].

### Measures

#### Physical activity measures

Participants completed two questions on the C_q_ regarding their daily number of minutes of both vigorous and moderate physical activity. Vigorous physical activity was defined as activities such as “jogging, team sports, fast dancing, and any other physical activities that increase your heart rate and make you breathe hard and sweat.” Moderate physical activity was defined as “lower intensity activities such as walking, biking to school, and recreational swimming.” Students could select the number of hours and minutes they engaged in each intensity level of physical activity for each day of the week. Based on responses to these questions, a continuous measure of average daily moderate to vigorous physical activity (MVPA) was calculated. Additionally, a binary measure of whether participants met the minimum Canadian physical activity guidelines of at least 60 minutes of MVPA per day was utilized. The COMPASS physical activity measures were found to have acceptable test-retest reliability and criterion validity [40]. While the correlation between self-report and accelerometer measures were low to modest, the results are comparable to most other studies using accelerometers to validate self-report physical activity [40].

Participants were also asked about their participation in intramural, varsity and league sports. To determine intramural sports participation participants were asked “Do you participate in before-school, noon hour, or after-school physical activities organized by your school?” To determine varsity sports participation participants were asked “Do you participate in competitive school sports teams that compete against other schools?” To determine league sports participation, participants were asked “Do you participate in league or team sports outside of school?” Participants could choose their response from: “Yes, No, or None offered at my school.”

#### Academic achievement measures

To measure academic achievement, participants were asked questions about their most recent Math and English grades with the questions “In your current or most recent Math course, what is your approximate overall mark?” and “In your current or most recent English course, what is your approximate overall mark?” Response options were: Less than 50%, 50%-59%, 60% - 69%, 70%-79%, 80%-89%, and “90% - 100%.” Grade categories were treated as continuous variables ranging from 1 to 6 with 1 indicating “Less than 50%” to 6 indicating “90% - 100%.” The higher the percentage indicated greater achievement, with 100% indicating a perfect grade and 50% signifying a passing grade.

#### Control variables

Participants provided demographic information on grade, sex, ethnicity and weekly spending money (as a proxy for socioeconomic status). To assess ethnicity, participants were asked “How would you describe yourself,” and could select any number of response options of “White”, “Black”, “Asian”, “Aboriginal (First Nations, Metis, Inuit)”, “Latin American/Hispanic” and “Other”. Due to sample sizes, response options were collapsed to “White”, “Asian”, “Other” and “Mixed”. To assess socioeconomic status, participants were asked “About how much money do you usually get each week to spend on yourself or save?” Response options ranged from “Zero” to “More than $100”, as well as “I don’t know”.

Additional control variables included participants’ perceived importance of grades, time spent sleeping, and estimated time spent doing homework. To measure perceived importance of grades, participants were asked their agreement with the statement “Getting good grades is important to me”. Response options were collapsed into “Strongly agree”, “Agree” and “Disagree/Strongly Disagree”. Time spent sleeping and doing homework were based on answers to the question “How much time per day do you usually spend doing the following activities?” and were measured in minutes per day.

### Statistical analysis

Descriptive statistics were calculated for all variables in the analysis at baseline (year 2), as well as academic achievement variables at follow-up (year 4). Data was included from two-year time points to maximize the sample size. Chi-square and t-tests were calculated in order to examine differences between the samples at baseline and follow-up (excluding missing values, sex, school grade and ethnicity), as well as those included and excluded at baseline (excluding missing values). Effect sizes were calculated for the relationship between baseline physical activity and grade at follow-up using correlations and Cohen’s *f* as effect size estimates (for raw and covariate-adjusted effects, respectively).

Sequential linear mixed models with random intercept, and multinomial ordinal generalized estimating equations (GEE) models were used to examine the association between baseline physical activity and sports participation on both academic outcomes (I.e., self-report English grades and self-reported Math grades) at follow-up. All models controlled for baseline school grade, sex, ethnicity, weekly spending money, perceived importance of grades, time spent sleeping and time spent doing homework, as well as corresponding baseline Math/English grade. Baseline Math and English grade was included as a control variable in order to isolate any prospective impact of physical activity on year 4 grades. Models also accounted for school-level clustering, with the assumption that students from the same school will be more alike than students from different schools. However, equivalent non-clustered models were used to calculate Cohen’s *f* effect sizes. All analyses were conducted using the statistical software SAS 9.4. The procedure PROC MIXED was used for linear models and PROC GENMOD was used for the logistic GEE model. Statistical significance was set a *p* < .05 for all analyses.

## Results

### Sample characteristics

Baseline descriptive information, including the demographic, academic and physical activity characteristics of the longitudinal sample (*N* = 9,898), as well as the comparison of the baseline and follow-up samples can be found in Table 1. At year 2, 52% of the participants met the national physical activity guidelines (60 minutes/day), and 53% of participants met the guidelines at year 4. In addition, participants completed a mean 122.1 minutes (± 81.3) average daily MVPA at baseline, and a mean 114.3 minutes (± 84.5) at follow-up. In terms of academic achievement, the majority of participants received Math and English grades in the 80–89% range (33% and 40% respectively) at baseline. This was consistent with the majority at year 4 follow-up (Math 29% and English 42%; Table 2).

**Table 1:** Baseline descriptive characteristics and comparisons of 9898 linked participants from the COMPASS study: Ontario and Alberta, Canada (2013-2014/2015-2016).

		Baseline (2013–2014)		Follow-up (2015–2016)		Comparison between Baseline and Follow-up			
**Variable**	**LevelsE**	**n**	**%**	**n**	**%**	**Chi-sq**	**df**	**p**	**Cramer’s V**
Grade	9	5110	52%	0	0%	-	-	-	-
	10	4466	45%	70	1%				
	11	320	3%	5057	51%				
	12	2	0%	4745	48%				
	Missing	-	-	26	0%				
Sex	Female	5226	53%	5197	53%	-	-	-	-
	Male	4672	47%	4630	47%				
	Missing	-	-	71	1%				
Ethnicity	White	7690	78%	7573	77%	-	-	-	-
	Asian	520	5%	550	6%				
	Other	992	10%	1104	11%				
	Mixed	696	7%	640	6%				
	Missing	-	-	31	0%				
Spending Money	Zero	1998	20%	1196	12%	26.931	7	< .0001	0.369
	$1 to $5	914	9%	389	4%				
	$6 to $10	1117	11%	456	5%				
	$11 to $20	1773	18%	1016	10%				
	$21 to $40	1220	12%	1134	11%				
	$41 to $100	981	10%	1788	18%				
	More than $100	597	6%	2899	29%				
	I don’t know	1298	13%	985	10%				
	Missing	n/a		35	0%	-	-	-	-
Importance of Grades	Strongly Agree	6003	61%	5637	57%	0.535	2	< .0001	0.052
	Agree	3568	36%	3671	37%				
	Disagree/Strongly Disagree	327	3%	512	5%				
	Missing	n/a		78	1%	-	-	-	-
Intramural Sports Participation	No	5908	60%	6177	62%	0.173	1	< .0001	-0.030
	Yes	3990	40%	3695	37%				
	Missing	n/a		26	0%	-	-	-	-
Varsity Sports Participation	No	5456	55%	5820	59%	23.350	1	< .0001	-0.039
	Yes	4442	45%	4055	41%				
	Missing	n/a		23	0%	-	-	-	-
League Sports Participation	No	4160	42%	5528	56%	390.883	1	< .0001	-0.141
	Yes	5738	58%	4327	44%				
	Missing	n/a		43	0%	-	-	-	-
English Grades	Less than 50%	67	1%	81	1%	21.039	5	0.0008	0.033
	50% - 59%	334	3%	377	4%				
	60% - 69%	1022	10%	967	10%				
	70% - 79%	3082	31%	2839	29%				
	80% - 89%	4005	40%	4156	42%				
	90% - 100%	1388	14%	1478	15%				
Math Grades	Less than 50%	184	2%	217	2%	70.841	5	< .0001	0.060
	50% - 59%	680	7%	851	9%				
	60% - 69%	1206	12%	1348	14%				
	70% - 79%	2380	24%	2602	26%				
	80% - 89%	3256	33%	2918	29%				
	90% - 100%	2192	22%	1962	20%				
Meets PA guidelines	Yes	4779	48%	4614	47%	5.516	1	0.019	0.017
	No	5119	52%	5284	53%				
**Variable (minutes/day)**		**Mean**	**SD**	**Mean**	**SD**	**T-test**	**df**	**p**	**Hedges’ G**
Time Spent Sleeping		424.8	130.6	404.7	115.8	11.480	19776	< .0001	0.163
Time Spent Doing Homework		94.3	70.4	109.4	91.0	-13.050	19776	< .0001	-0.186
Average Daily MVPA		122.1	81.3	114.4	84.5	6.590	19794	< .0001	0.094

**Table 2:** Relationship between physical activity predictors and English grades: Ontario and Alberta, Canada (2013-2014/2015-2016).

	Model 1			Model 2- MVPA			Model 3- Meets Guidelines			Model 4- Sports Participation		
	Est.	S.E.	p-value	Est.	S.E.	p-value	Est.	S.E.	p-value	Est.	S.E.	p-value
Intercept												
	2.87	0.07	< .00	2.90	0.07	< .00	2.89	0.07	< .00	2.85	0.07	< .00
Average Daily MVPA (minutes) at Baseline												
				-0.00	0.00	0.03						
Meets PA Guidelines at Baseline												
No (ref)												
Yes							-0.05	0.02	0.00			
Intramural Sports Participation												
No												
Yes										0.05	0.02	0.02
Varsity Sports Participation												
No												
Yes										0.04	0.02	0.07
League Sports Participation												
No												
Yes										0.01	0.02	0.66

Estimates generated using linear regression mixed models. All models controlled for baseline school grade, sex, ethnicity, weekly spending money, perceived importance of grades, time spent sleeping and time spent doing homework, as well as corresponding baseline Math/English grade. Models also accounted for school-level clustering. The complete tables with all parameter estimates (including control variables) can be found in the S1 File.

Comparisons between samples included from year 2 (2013–2014) and year 4 (2015–2016) revealed significantly more spending money (*χ*^2^ = 26.931, *p* < .0001, Cramer’s *V* = .369), more time spend sleeping (*t* = 11.480, *p* < .0001, Hedge’s *G* = .163), less league sport participation (*χ*^2^ = 390.883, *p* < .0001, Cramer’s *V* = -.141), and less time spent doing homework (*t* = -13.050, *p* < .0001, Hedge’s *G* = -.186) at follow-up. For all other variables, significant associations exit, but with a trivial magnitude of association (Cramer’s *V* < .100 or Hedge’s *G* < .200).

A comparison of baseline characteristics of the included versus excluded populations was also performed (See S1 File). The included sample was significantly different that the excluded sample for all variables at baseline; however, in most cases the magnitude of difference was small (Cramer’s *V* < .100 or Hedge’s *G* < .200). The included sample reported a significantly higher importance of grades (*χ*^2^ = 448.156, *p* < .0001, Cramer’s *V* = .139), had higher English (*χ*^2^ = 406.111, *p* < .0001, Cramer’s *V* = .133) and Math grades (*χ*^2^ = 351.255, *p* < .0001, Cramer’s *V* = .123), had more minutes of average daily MVPA (*t* = 8.040, *p* < .0001, Hedge’s *G* = .106), and spent less time doing homework (*t* = -8.760, *p* < .0001, Hedge’s *G* = -.115).

### Physical activity and academic achievement

**Fig 1:**
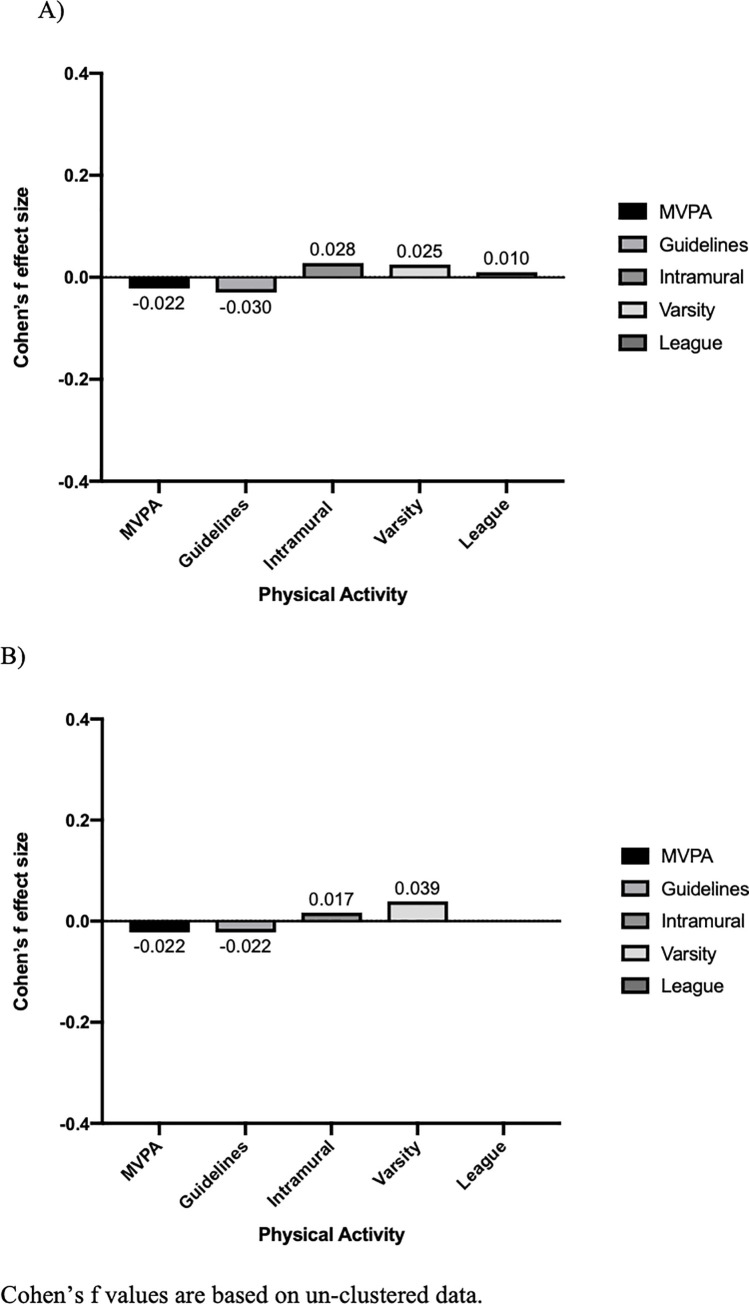
Cohen’s *f* effect sizes. The relationship between physical activity predictors and (A) English grades as well as (B) Math grades: Ontario and Alberta (2013-2014/2015-2016).

Zero-order Pearson correlation coefficients linking baseline average daily MVPA with subsequent academic achievement were statistically significant, but negative in direction and trivial in absolute magnitude (English: *r* = -.047, *p* < .000; Math: *r* = -.026, *p* = .009). This association is consistent with the covariate-adjusted linear regression models, which revealed a significant negative but trivial magnitude association between average daily MVPA at baseline and both academic outcomes at follow-up (Tables 2 and 3; English: *est* = -.000, *p* = .029; Math: *est* = -.000, *p* = .019). The Cohen’s *f* effect size for this relationship was as follows: English: *f* = -.022; Math: *f* = -.022 (Table 4). Fig 1 provides a graphical representation of the Cohen’s *f* values for all relationships between physical activity predictors and both English and Math achievement.

**Table 3:** Relationship between physical activity predictors and Math grades: Ontario and Alberta, Canada (2013-2014/2015-2016).

	Model 1			Model 2—MVPA			Model 3 –Meeting Guidelines			Model 4 –Sports Participation		
	est.	SE	*P*	est.	SE	*P*	est.	SE	*P*	est.	SE	*P*
Intercept												
	2.58	0.07	< .00	2.61	0.08	< .00	2.59	0.08	< .00	2.56	0.08	< .00
Average Daily MVPA (minutes) at Baseline												
				-0.00	0.00	0.02						
Meets PA Guidelines at Baseline												
No (ref)												
Yes							-0.05	0.02	0.03			
Intramural Sports Participation												
No (ref)												
Yes										0.02	0.03	0.57
Varsity Sports Participation												
No (ref)												
Yes										0.09	0.03	0.00
League Sports Participation												
No (ref)												
Yes										-0.01	0.03	0.67

Estimates generated using linear regression mixed models. All models controlled for baseline school grade, sex, ethnicity, weekly spending money, perceived importance of grades, time spent sleeping and time spent doing homework, as well as corresponding baseline Math/English grade. Models also accounted for school-level clustering. The complete tables with all parameter estimates (including control variables) can be found in the S1 File.

**Table 4:** Effect sizes for the relationship between physical activity predictors and academic outcomes: Ontario and Alberta, Canada (2013-2014/2015-2016).

	English Grade	Math Grade
Average Daily MVPA (minutes) at Baseline	-0.022	-0.022
Meets PA Guidelines at Baseline	-0.030	-0.022
Intramural Sports Participation	0.028	0.017
Varsity Sports Participation	0.025	0.039
League Sports Participation	0.010	-

Coefficients are Cohen’s *f* values based on un-clustered data.

Similar to average daily MVPA, the likelihood of better academic achievement was lower as a function of meeting the national physical activity guidelines (English: *est* = -.052, *p* = .004; Math: *est* = -.052, *p* = .028) in covariate-adjusted models; again however, the effect was trivial in absolute magnitude (English: *f* = - .030; Math: *f* = -.022).

Organized sport participation was significantly associated with higher grades in Math and English, but the magnitude of these associations approached zero (Tables 2 and 3; Fig 1). Overall, effect sizes for activity predictors academic outcomes were only about 30% or less of the threshold for a “small” effect (*f* = .14) by Cohen’s interpretive conventions.

Categorical analysis of baseline physical activity variables and both academic outcomes using multinomial ordinal GEE models are presented in Table 5. In general, the results were consistent with the continuous analysis. Complete tables with all parameter estimates can be found in S1 File.

**Table 5:** Categorical analysis of the physical activity predictors and academic outcomes: Ontario and Alberta, Canada (2013-2014/2015-2016).

	English Grades						Math Grades					
	Model 1- Meeting Guidelines			Model 2- MVPA			Model 3 –Meeting Guidelines			Model 4 –MVPA		
	est.	SE	*P*	est.	SE	*P*	est.	SE	*P*	est.	SE	*P*
Intercept												
1	-6.58	0.24	< .00	-6.54	0.24	< .00	-4.81	0.17	< .00	-4.77	0.16	< .00
2	-4.13	0.21	< .00	-4.10	0.21	< .00	-3.23	0.16	< .00	-3.19	0.16	< .00
3	-2.28	0.19	< .00	-2.24	0.19	< .00	-1.88	0.15	< .00	-1.84	0.15	< .00
4	-0.84	0.19	< .00	-0.81	0.19	< .00	-0.77	0.15	< .00	-0.72	0.15	< .00
5	-0.08	0.18	0.66	-0.04	0.18	0.82	-0.11	0.15	0.49	-0.06	0.15	0.69
6	1.04	0.18	< .00	1.08	0.18	< .00	1.02	0.16	< .00	1.07	0.16	< .00
Average Daily MVPA												
				0.00	0.00	< .00				0.00	0.00	< .00
Meets Guidelines at Baseline												
No (ref)												
Yes	-0.17	0.04	< .00				-0.14	0.04	0.00			
Intramural Sports Participation												
No (ref)												
Yes	0.13	0.06	0.02	0.14	0.06	0.01	0.08	0.05	0.16	0.09	0.05	0.10
Varsity Sports Participation												
No (ref)												
Yes	0.14	0.05	0.01	0.15	0.05	0.01	0.20	0.04	< .00	0.21	0.04	< .00
League Sports Participation												
No (ref)												
Yes	0.03	0.05	0.55	0.05	0.05	0.32	0.00	0.05	0.92	0.02	0.05	0.67

Estimates generated using multinomial ordinal Generalized Estimating Equations (GEE) models. All models controlled for baseline school grade, sex, ethnicity, weekly spending money, perceived importance of grades, time spent sleeping and time spent doing homework, as well as corresponding baseline Math/English grade. Models also accounted for school-level clustering. The complete tables with all parameter estimates (including control variables) can be found in the S1 File.

## Discussion

The present investigation examined the magnitude and direction of the relationship between three indicators of physical activity (minutes of MVPA, percent achieving national physical activity guidelines, and organized sports participation) and two indicators of academic achievement (English and Math grades) in adolescents. Both continuous and categorical analyses of the variables found near-zero magnitude associations between physical activity at baseline and self-reported academic achievement at follow-up, after controlling for confounders. Similarly, meeting the national physical activity guidelines at baseline had no substantive benefit in relation to academic achievement at follow-up. In short, there was little support for a hypothesized net benefit of physical activity on academic achievement.

Of equal importance, no evidence of adverse impact of physical activity on academic achievement was observed, indicating that higher levels of physical activity may have no substantial adverse effect overall on academic achievement. The current study effectively suggests that any concerns regarding the time competition between physical activity and academic achievement among youth may be, on the whole, not well-founded. These results are meaningful when the sizable nature of this study is considered, as with the use of two continuous measures of academic achievement, which utilize all the variability within the dataset. In addition, all parameter estimates were highly precise (i.e., narrow confidence intervals) and generalizable to the larger population from which the sample was drawn.

This study has found little evidence of benefit from MVPA or from meeting the national physical activity guidelines on either indicator of academic achievement. Previous literature has hypothesized a positive relationship between the two variables as it is thought that the brain health benefits observed from exercise could contribute to increased academic achievement, as regions associated with the observed “brain benefits” of exercise are also important for academic attainment [9, 10, 12, 13, 21]. These findings are corroborated by a recent analysis on this topic using COMPASS data 2015/16 and 2016/17 waves and categorical measures of achievement, which found trivial effects of meeting the physical activity guidelines [29] and by a recent investigation that found evidence of a brain health benefit from accelerometer-assessed physical activity, but no significant associations between physical activity and academic achievement outcomes [20]. Previous experimental studies have found positive effects of exercise on achievement test performance [8, 42], and so there is an opportunity for future experimental research into the brain-benefit vs. time-competition hypotheses.

A reliable but trivial (*f* < .03) benefit of varsity sport participation was observed in relation to academic achievement. The impact of sport participation was not included in the prior investigation into the relationship between physical activity and academic achievement that utilized COMPASS data [29]. These findings are unique to this analysis, however they may or may not be of practical importance as it may arise solely via minimum academic requirements for participation in varsity athletics, for example. However, it is also the case that the more complex social environment offered by sport participation (compared to unstructured physical activity alone) could benefit brain development [43]. In addition, the social benefits, such as the formation of formal or informal academic assistance on school-based teams that do not work through the brain could potentially lead to slightly enhanced academic achievement that has nothing to do with the brain *per se*. Finally, it is also possible that adolescents could experience other mental, social or physical health challenges that adversely impact both academic and sport participation, producing a similar pattern to what has been observed in this study. Further research will be required in order to disentangle these possibilities.

The sample size determination for the COMPASS study was based on a number of analyses that were not part of the current paper, mostly involving ensuring sufficient power for sub-group analysis (i.e., within age group, within sex, within grade level), and it is not directly relevant to the current paper. With a sample size of nearly 10,000, we have more than sufficient sample size for all of the current analyses, and so unstable parameter estimates, or lack of statistical power are not of concern. This is evidenced by the fact that even trivial effect sizes (Cohen’s *f* < .05) are statistically significant. Notably, the current findings provide a good example of the disjunction between effect size and statistical significance in large samples. Here we have documented trivial magnitude effects that are highly statistically significant simply because of the large sample size. Given the very narrow confidence intervals around our estimates of association, we can be reasonably confident in the precision of our estimates of the relationship magnitude between physical activity and academic achievement. This is an instance where the effect magnitude and confidence intervals are the focus of discussion rather than *p*-value [44].

This study utilizes self-reported measures of both physical activity and academic achievement. The COMPASS student questionnaire was designed to measure a wide variety of health behaviours from a large population of adolescents [38]. The large longitudinal sample allowed for greater predictions of the parameter estimates, and the inclusion of multiple physical activity and academic achievement variables allowed for a comprehensive analysis; however, the use of self-reported measures reduced the precision of measurement. The use of accelerometers and objective measures of academic achievement (e.g., academic testing or school grades) could increase precision of measurement of vigorous, and especially moderate, physical activity. However, logistics of using accelerometers and academic testing in studies involving sample sizes of this nature are generally prohibitive. In the future, subsets of participants from COMPASS could be assessed using accelerometer based physical activity.

The strengths of the current investigation include the use of a large longitudinal sample and high generalizability. Treating achievement outcomes as continuous variables also allowed for the full use of the variability within the data. In addition, the COMPASS survey is a reliable and valid measure concordant with national surveillance tools and public health guidelines, which also lends to the credibility of the findings [40]. The main limitation of this study is the use of self-reported physical activity and academic achievement measures, both of which may be subject to social desirability bias and contribute to systematic error in measurement. The use of self-reported measures also contributed missing data, which limited the available sample size for this study to a certain extent. Such influences may have impacted the magnitude and significance of the relationships examined. While the magnitude of association was small, there were some differences reported between participants at baseline and follow-up, as well as those included and excluded from analysis, which could have impacted the findings. Future studies should include the use of accelerometers as a more objective measure of physical activity, and record the actual grades received in English and Math as opposed to collecting academic achievement as a self-reported measure. It would also be beneficial to examine the longer-term exercise and academic trends over the lifespan to provide an even more accurate measure of the effect.

## Conclusions

The present study sought to estimate the magnitude of the association between physical activity and academic achievement in a large sample of adolescents. Findings suggested a small but trivial magnitude of associations between all indicators of physical activity and the academic achievement outcomes. While the current results do not support the assumed academic benefits of physical activity, these results are consistent with another longitudinal investigation into the topic using COMPASS data [41]. Furthermore, at least one additional small-scale study does appear to support the notion that brain health benefits of physical activity are realized in adolescents, despite unclear translation of such brain health benefits into overt academic achievement benefits [15]. Future large scale observational and experimental studies should employ more stringent measures of academic achievement (e.g., the actual grades received in English and Math class) and physical activity in order to examine the possibility of mutual offsetting of time cost and brain benefit in relation to physical activity in the academic context. From a policy perspective, the results advocate for the inclusion of physical activity programming based on the demonstrated mental and physical health benefits, rather than on expectation of net academic benefit. Public health initiatives or policies promoting physical activity in adolescents can do so without fear of adversely impacting academic achievement.

## Supporting information

S1 File Baseline descriptive characteristics and categorical analyses between physical activity predictors and grades. (DOCX)
