# Correction: An examination of the prospective association between physical activity and academic achievement in youth at the population level

**DOI:** 10.1371/journal.pone.0259618

**Published:** 2021-11-01

**Authors:** Mia Papasideris, Scott T. Leatherdale, Kate Battista, Peter A. Hall

Arbona (2005) was incorrectly included as reference 15. As a result, all subsequent references are misnumbered. References 16–44 should be references 15–43.

The third sentence of the Conclusions section should have cited reference 28 (29 in the original article) instead of 40 (41 in the original article).

The correct sentence should read: While the current results do not support the assumed academic benefits of physical activity, these results are consistent with another longitudinal investigation into the topic using COMPASS data [28].

The fourth sentence of the Conclusions section should have cited reference 19 (20 in the original article) instead of 15 (the removed reference).

The correct sentence should read: Furthermore, at least one additional small-scale study does appear to support the notion that brain health benefits of physical activity are realized in adolescents, despite unclear translation of such brain health benefits into overt academic achievement benefits [19].
