# Supplementary material for: An examination of the prospective association between physical activity and academic achievement in youth at the population level
Source: PLoS One. 2021 Jun 11;16(6):e0253142. doi: 10.1371/journal.pone.0253142 (PMC8195429; doi:10.1371/journal.pone.0253142)
Supplement: S1 File — (DOCX) [file pone.0253142.s001.docx]

**S1 File. Baseline descriptive characteristics and categorical analyses between physical activity predictors and grades**

**Table 1. Baseline descriptive characteristics and comparisons of included and excluded participants from the COMPASS Study: Ontario and Alberta, Canada (2013-2014/2015-2016)**

|  |  | **Excluded (n = 14034)** | | | **Included (n = 9898)** | | | **Comparison between included and excluded** | | | |
| --- | --- | --- | --- | --- | --- | --- | --- | --- | --- | --- | --- |
| **Variable** | **Levels** | **n** | | **%** | **n** |  | **%** | **Chi-sq** | **df** | **p** | **Cramer's V** |
| Grade | 9 | 6683 |  | 48% | 5110 |  | 52% | 537.412 | 3 | <.0001 | 0.150 |
|  | 10 | 7351 |  | 52% | 4466 |  | 45% |  |  |  |  |
|  | 11 | 0 |  | 0% | 320 |  | 3% |  |  |  |  |
|  | 12 | 0 |  | 0% | 2 |  | 0% |  |  |  |  |
|  | Missing | 0 |  | 0% | 0 |  | 0% | - | - | - | - |
| Sex | Female | 6528 |  | 47% | 5226 |  | 53% | 79.483 | 1 | <.0001 | -0.058 |
|  | Male | 7380 |  | 53% | 4672 |  | 47% |  |  |  |  |
|  | Missing | 126 |  | 1% | 0 |  | 0% | - | - | - | - |
| Ethnicity | White | 10180 |  | 73% | 7690 |  | 78% | 146.158 | 3 | <.0001 | 0.078 |
|  | Asian | 573 |  | 4% | 520 |  | 5% |  |  |  |  |
|  | Other | 2089 |  | 15% | 992 |  | 10% |  |  |  |  |
|  | Mixed | 1061 |  | 8% | 696 |  | 7% |  |  |  |  |
|  | Missing | 131 |  | 1% | 0 |  | 0% | - | - | - | - |
| Spending Money | Zero | 2602 |  | 19% | 1998 |  | 20% | 121.234 | 7 | <.0001 | 0.071 |
|  | $1 to $5 | 1140 |  | 8% | 914 |  | 9% |  |  |  |  |
|  | $6 to $10 | 1311 |  | 9% | 1117 |  | 11% |  |  |  |  |
|  | $11 to $20 | 2374 |  | 17% | 1773 |  | 18% |  |  |  |  |
|  | $21 to $40 | 1853 |  | 13% | 1220 |  | 12% |  |  |  |  |
|  | $41 to $100 | 1422 |  | 10% | 981 |  | 10% |  |  |  |  |
|  | More than $100 | 1302 |  | 9% | 597 |  | 6% |  |  |  |  |
|  | I don't know | 1858 |  | 13% | 1298 |  | 13% |  |  |  |  |
|  | Missing | 172 |  | 1% | 0 |  | 0% | - | - | - | - |
| Importance of Grades | Strongly Agree | 6448 |  | 46% | 6003 |  | 61% | 448.156 | 2 | <.0001 | 0.139 |
|  | Agree | 5806 |  | 41% | 3568 |  | 36% |  |  |  |  |
|  | Disagree/ Strongly Disagree | 1067 |  | 8% | 327 |  | 3% |  |  |  |  |
|  | Missing | 713 |  | 5% | 0 |  | 0% | - | - | - | - |
| Intramural Sports Participation | No | 8563 |  | 61% | 5908 |  | 60% | 18.156 | 1 | <.0001 | 0.028 |
|  | Yes | 5154 |  | 37% | 3990 |  | 40% |  |  |  |  |
|  | Missing | 317 |  | 2% | 0 |  | 0% | - | - | - | - |
| Varsity Sports Participation | No | 7943 |  | 57% | 5456 |  | 55% | 18.264 | 1 | <.0001 | 0.028 |
|  | Yes | 5772 |  | 41% | 4442 |  | 45% |  |  |  |  |
|  | Missing | 319 |  | 2% | 0 |  | 0% | - | - | - | - |
| League Sports Participation | No | 6483 |  | 46% | 4160 |  | 42% | 66.667 | 1 | <.0001 | 0.053 |
|  | Yes | 7197 |  | 51% | 5738 |  | 58% |  |  |  |  |
|  | Missing | 354 |  | 3% | 0 |  | 0% | - | - | - | - |
| English Grades | Less than 50% | 306 |  | 2% | 67 |  | 1% | 406.111 | 5 | <.0001 | 0.133 |
|  | 50% - 59% | 908 |  | 6% | 334 |  | 3% |  |  |  |  |
|  | 60% - 69% | 1889 |  | 13% | 1022 |  | 10% |  |  |  |  |
|  | 70% - 79% | 4079 |  | 29% | 3082 |  | 31% |  |  |  |  |
|  | 80% - 89% | 4444 |  | 32% | 4005 |  | 40% |  |  |  |  |
|  | 90% - 100% | 1458 |  | 10% | 1388 |  | 14% |  |  |  |  |
|  | Missing | 950 |  | 7% | 0 |  | 0% | - | - | - | - |
| Math Grades | Less than 50% | 571 |  | 4% | 184 |  | 2% | 351.255 | 5 | <.0001 | 0.123 |
|  | 50% - 59% | 1393 |  | 10% | 680 |  | 7% |  |  |  |  |
|  | 60% - 69% | 2024 |  | 14% | 1206 |  | 12% |  |  |  |  |
|  | 70% - 79% | 3284 |  | 23% | 2380 |  | 24% |  |  |  |  |
|  | 80% - 89% | 3660 |  | 26% | 3256 |  | 33% |  |  |  |  |
|  | 90% - 100% | 2285 |  | 16% | 2192 |  | 22% |  |  |  |  |
|  | Missing | 817 |  | 6% | 0 |  | 0% | - | - | - | - |
| Meets PA guidelines | Yes | 6993 |  | 50% | 4779 |  | 48% | 33.967 | 1 | <.0001 | 0.038 |
|  | No | 6418 |  | 46% | 5119 |  | 52% |  |  |  |  |
|  | Missing | 623 |  | 4% | 0 |  | 0% | - | - | - | - |
| **Variable (minutes/day)** | | **Missing** | **Mean** | **Std** | **Missing** | **Mean** | **Std** | **T-test** | **df** | **p** | **Hedges' G** |
| Time Spent Sleeping | | 72.000 | 414.199 | 143.463 | 0.000 | 424.799 | 130.598 | -5.830 | 23858.000 | <.0001 | -0.077 |
| Time Spent Doing Homework | | 72.000 | 85.618 | 78.127 | 0.000 | 94.252 | 70.424 | -8.760 | 23858.000 | <.0001 | -0.115 |
| Average Daily MVPA | | 623.000 | 131.512 | 93.045 | 0.000 | 122.114 | 81.312 | 8.040 | 23307.000 | <.0001 | 0.106 |

**Table 2.** **Relationship between Physical Activity Predictors and English Grades: Ontario and Alberta, Canada (2013-2014/2015-2016)**

|  | Model 1 | | | Model 2- MVPA | | | Model 3- Meets Guidelines | | | Model 4- Sports Participation | | |
| --- | --- | --- | --- | --- | --- | --- | --- | --- | --- | --- | --- | --- |
|  | Est. | S.E. | p-value | Est. | S.E. | p-value | Est. | S.E. | p-value | Est. | S.E. | p-value |
| Intercept | | | | | | | | | | | | |
|  | 2.87 | 0.07 | <.00 | 2.90 | 0.07 | <.00 | 2.89 | 0.07 | <.00 | 2.85 | 0.07 | <.00 |
| Grade | | | | | | | | | | | | |
| 9 (ref) |  |  |  |  |  |  |  |  |  |  |  |  |
| 10 | 0.09 | 0.02 | <.00 | 0.09 | 0.02 | <.00 | 0.09 | 0.02 | <.00 | 0.09 | 0.02 | <.00 |
| 11 | 0.15 | 0.05 | 0.00 | 0.15 | 0.05 | 0.00 | 0.15 | 0.05 | 0.00 | 0.15 | 0.05 | 0.00 |
| 12 | 0.22 | 0.62 | 0.72 | 0.22 | 0.62 | 0.72 | 0.22 | 0.62 | 0.72 | 0.22 | 0.62 | 0.72 |
| Sex | | | | | | | | | | | | |
| Female (ref) |  |  |  |  |  |  |  |  |  |  |  |  |
| Male | -0.25 | 0.02 | <.00 | -0.24 | 0.02 | <.00 | -0.24 | 0.02 | <.00 | -0.25 | 0.02 | <.00 |
| Ethnicity | | | | | | | | | | | | |
| White (ref) |  |  |  |  |  |  |  |  |  |  |  |  |
| Asian | -0.03 | 0.04 | 0.52 | -0.03 | 0.04 | 0.46 | -0.03 | 0.04 | 0.46 | -0.02 | 0.04 | 0.64 |
| Other | -0.15 | 0.03 | <.00 | -0.15 | 0.03 | <.00 | -0.16 | 0.03 | <.00 | -0.15 | 0.03 | <.00 |
| Mixed | -0.07 | 0.04 | 0.04 | -0.07 | 0.03 | 0.04 | -0.07 | 0.03 | 0.04 | -0.07 | 0.04 | 0.05 |
| Spending Money | | | | | | | | | | | | |
| Zero (ref) |  |  |  |  |  |  |  |  |  |  |  |  |
| $1 to $5 | -0.05 | 0.04 | 0.17 | -0.05 | 0.04 | 0.19 | -0.05 | 0.04 | 0.19 | -0.05 | 0.04 | 0.16 |
| $6 to $10 | -0.03 | 0.03 | 0.29 | -0.03 | 0.03 | 0.34 | -0.03 | 0.03 | 0.34 | -0.04 | 0.03 | 0.27 |
| $11 to $20 | -0.01 | 0.03 | 0.72 | -0.01 | 0.03 | 0.83 | -0.01 | 0.03 | 0.84 | -0.02 | 0.03 | 0.56 |
| $21 to $40 | 0.00 | 0.03 | 0.88 | 0.00 | 0.03 | 0.97 | 0.00 | 0.03 | 0.98 | -0.01 | 0.03 | 0.66 |
| $41 to $100 | 0.01 | 0.03 | 0.78 | 0.02 | 0.03 | 0.59 | 0.02 | 0.03 | 0.60 | 0.00 | 0.03 | 0.93 |
| More than $100 | -0.04 | 0.04 | 0.33 | -0.03 | 0.04 | 0.50 | -0.03 | 0.04 | 0.47 | -0.05 | 0.04 | 0.23 |
| I don't know | -0.02 | 0.03 | 0.44 | -0.02 | 0.03 | 0.53 | -0.02 | 0.03 | 0.54 | -0.03 | 0.03 | 0.35 |
| Importance of Grades | | | | | | | | | | | | |
| Strongly Agree (ref) |  |  |  |  |  |  |  |  |  |  |  |  |
| Agree | -0.25 | 0.02 | <.00 | -0.25 | 0.02 | <.00 | -0.25 | 0.02 | <.00 | -0.24 | 0.02 | <.00 |
| Disagree/  Strongly Disagree | -0.41 | 0.05 | <.00 | -0.42 | 0.05 | <.00 | -0.41 | 0.05 | <.00 | -0.39 | 0.05 | <.00 |
| Grade at Baseline | | | | | | | | | | | | |
|  | 0.39 | 0.01 | <.00 | 0.39 | 0.01 | <.00 | 0.39 | 0.01 | <.00 | 0.38 | 0.01 | <.00 |
| Time Spent Sleeping (minutes/day) | | | | | | | | | | | | |
|  | 0.00 | 0.00 | 0.02 | 0.00 | 0.00 | 0.01 | 0.00 | 0.00 | 0.01 | 0.00 | 0.00 | 0.02 |
| Time Spent Doing Homework (minutes/day) | | | | | | | | | | | | |
|  | 0.00 | 0.00 | 0.08 | 0.00 | 0.00 | 0.06 | 0.00 | 0.00 | 0.06 | 0.00 | 0.00 | 0.11 |
| Average Daily MVPA (minutes) at Baseline | | | | | | | | | | | | |
|  |  |  |  | -0.00 | 0.00 | 0.03 |  |  |  |  |  |  |
| Meets PA Guidelines at Baseline | | | | | | | | | | | | |
| No (ref) |  |  |  |  |  |  |  |  |  |  |  |  |
| Yes |  |  |  |  |  |  | -0.05 | 0.02 | 0.00 |  |  |  |
| Intramural Sports Participation | | | | | | | | | | | | |
| No |  |  |  |  |  |  |  |  |  |  |  |  |
| Yes |  |  |  |  |  |  |  |  |  | 0.05 | 0.02 | 0.02 |
| Varsity Sports Participation | | | | | | | | | | | | |
| No |  |  |  |  |  |  |  |  |  |  |  |  |
| Yes |  |  |  |  |  |  |  |  |  | 0.04 | 0.02 | 0.07 |
| League Sports Participation | | | | | | | | | | | | |
| No |  |  |  |  |  |  |  |  |  |  |  |  |
| Yes |  |  |  |  |  |  |  |  |  | 0.01 | 0.02 | 0.66 |

Estimates generated using linear regression mixed models.

**Table 3.** **Relationship between Physical Activity Predictors and Math Grades: Ontario and Alberta, Canada (2013-2014/2015-2016)**

|  | Model 1 | | | Model 2 - MVPA | | | Model 3 – Meeting Guidelines | | | Model 4 – Sports Participation | | |
| --- | --- | --- | --- | --- | --- | --- | --- | --- | --- | --- | --- | --- |
|  | est. | SE | *P* | est. | SE | *P* | est. | SE | *P* | est. | SE | *P* |
| Intercept | | | | | | | | | | | | |
|  | 2.58 | 0.07 | <.00 | 2.61 | 0.08 | <.00 | 2.59 | 0.08 | <.00 | 2.56 | 0.08 | <.00 |
| Grade |  |  |  |  |  |  |  |  |  |  |  |  |
| 9 (ref) |  |  |  |  |  |  |  |  |  |  |  |  |
| 10 | 0.05 | 0.02 | 0.03 | 0.05 | 0.02 | 0.06 | 0.05 | 0.02 | 0.05 | 0.05 | 0.02 | 0.04 |
| 11 | 0.09 | 0.07 | 0.17 | 0.09 | 0.07 | 0.20 | 0.09 | 0.07 | 0.18 | 0.09 | 0.07 | 0.17 |
| 12 | 0.05 | 0.80 | 0.95 | 0.04 | 0.80 | 0.96 | 0.05 | 0.80 | 0.95 | 0.05 | 0.80 | 0.95 |
| Sex |  |  |  |  |  |  |  |  |  |  |  |  |
| Female (ref) |  |  |  |  |  |  |  |  |  |  |  |  |
| Male | -0.10 | 0.02 | <.00 | -0.09 | 0.02 | 0.00 | -0.09 | 0.02 | 0.00 | -0.10 | 0.02 | <.00 |
| Ethnicity |  |  |  |  |  |  |  |  |  |  |  |  |
| White (ref) |  |  |  |  |  |  |  |  |  |  |  |  |
| Asian | 0.06 | 0.05 | 0.23 | 0.06 | 0.05 | 0.28 | 0.06 | 0.05 | 0.26 | 0.07 | 0.05 | 0.19 |
| Other | -0.13 | 0.04 | 0.00 | -0.13 | 0.04 | 0.00 | -0.13 | 0.04 | 0.00 | -0.13 | 0.04 | 0.00 |
| Mixed | -0.05 | 0.05 | 0.32 | -0.05 | 0.05 | 0.32 | -0.05 | 0.05 | 0.31 | -0.04 | 0.05 | 0.33 |
| Spending Money |  |  |  |  |  |  |  |  |  |  |  |  |
| Zero (ref) |  |  |  |  |  |  |  |  |  |  |  |  |
| $1 to $5 | -0.06 | 0.05 | 0.17 | -0.06 | 0.05 | 0.19 | -0.06 | 0.05 | 0.18 | -0.06 | 0.05 | 0.17 |
| $6 to $10 | -0.05 | 0.04 | 0.25 | -0.05 | 0.04 | 0.29 | -0.05 | 0.04 | 0.28 | -0.05 | 0.04 | 0.25 |
| $11 to $20 | -0.04 | 0.04 | 0.28 | -0.03 | 0.04 | 0.36 | -0.04 | 0.04 | 0.34 | -0.04 | 0.04 | 0.24 |
| $21 to $40 | -0.07 | 0.04 | 0.09 | -0.06 | 0.04 | 0.13 | -0.07 | 0.04 | 0.12 | -0.08 | 0.04 | 0.06 |
| $41 to $100 | -0.02 | 0.04 | 0.71 | 0.00 | 0.05 | 0.92 | -0.01 | 0.05 | 0.86 | -0.03 | 0.05 | 0.55 |
| More than $100 | -0.09 | 0.05 | 0.10 | -0.07 | 0.05 | 0.19 | -0.08 | 0.05 | 0.15 | -0.10 | 0.05 | 0.08 |
| I don't know | 0.01 | 0.04 | 0.76 | 0.02 | 0.04 | 0.64 | 0.02 | 0.04 | 0.67 | 0.01 | 0.04 | 0.82 |
| Importance of Grades | | | | | | | | | | | | |
| Strongly Agree (ref) |  |  |  |  |  |  |  |  |  |  |  |  |
| Agree | -0.20 | 0.03 | <.00 | -0.20 | 0.03 | <.00 | -0.20 | 0.03 | <.00 | -0.20 | 0.03 | <.00 |
| Disagree/Strongly Disagree | -0.27 | 0.07 | <.00 | -0.28 | 0.07 | <.00 | -0.28 | 0.07 | <.00 | -0.26 | 0.07 | 0.00 |
| Grades at baseline | | | | | | | | | | | | |
|  | 0.39 | 0.01 | <.00 | 0.39 | 0.01 | <.00 | 0.39 | 0.01 | <.00 | 0.39 | 0.01 | <.00 |
| Time Spent Sleeping (minutes/day) | | | | | | | | | | | | |
|  | 0.00 | 0.00 | 0.00 | 0.00 | 0.00 | 0.00 | 0.00 | 0.00 | 0.00 | 0.00 | 0.00 | 0.00 |
| Time Spent Doing Homework (minutes/day) | | | | | | | | | | | | |
|  | 0.00 | 0.00 | 0.61 | 0.00 | 0.00 | 0.52 | 0.00 | 0.00 | 0.55 | 0.00 | 0.00 | 0.67 |
| Average Daily MVPA (minutes) at Baseline | | | | | | | | | | | | |
|  |  |  |  | -0.00 | 0.00 | 0.02 |  |  |  |  |  |  |
| Meets PA Guidelines at Baseline | | | | | | | | | | | | |
| No (ref) |  |  |  |  |  |  |  |  |  |  |  |  |
| Yes |  |  |  |  |  |  | -0.05 | 0.02 | 0.03 |  |  |  |
| Intramural Sports Participation | | | | | | | | | | | | |
| No (ref) |  |  |  |  |  |  |  |  |  |  |  |  |
| Yes |  |  |  |  |  |  |  |  |  | 0.02 | 0.03 | 0.57 |
| Varsity Sports Participation | | | | | | | | | | | | |
| No (ref) |  |  |  |  |  |  |  |  |  |  |  |  |
| Yes |  |  |  |  |  |  |  |  |  | 0.09 | 0.03 | 0.00 |
| League Sports Participation | | | | | | | | | | | | |
| No (ref) |  |  |  |  |  |  |  |  |  |  |  |  |
| Yes |  |  |  |  |  |  |  |  |  | -0.01 | 0.03 | 0.67 |

Estimates generated using linear regression mixed models

**Table 4**. **Categorical Analysis of the Physical Activity Predictors and Academic Outcomes: Ontario and Alberta, Canada (2013-2014/2015-2016)**

|  | English Grades | | | | | | Math Grades | | | | | |
| --- | --- | --- | --- | --- | --- | --- | --- | --- | --- | --- | --- | --- |
|  | Model 1- Meeting Guidelines | | | Model 2- MVPA | | | Model 3 – Meeting Guidelines | | | Model 4 – MVPA | | |
|  | est. | SE | *P* | est. | SE | *P* | est. | SE | *P* | est. | SE | *P* |
| Intercept | | | | | | | | | | | | |
| 1 | -6.58 | 0.24 | <.00 | -6.54 | 0.24 | <.00 | -4.81 | 0.17 | <.00 | -4.77 | 0.16 | <.00 |
| 2 | -4.13 | 0.21 | <.00 | -4.10 | 0.21 | <.00 | -3.23 | 0.16 | <.00 | -3.19 | 0.16 | <.00 |
| 3 | -2.28 | 0.19 | <.00 | -2.24 | 0.19 | <.00 | -1.88 | 0.15 | <.00 | -1.84 | 0.15 | <.00 |
| 4 | -0.84 | 0.19 | <.00 | -0.81 | 0.19 | <.00 | -0.77 | 0.15 | <.00 | -0.72 | 0.15 | <.00 |
| 5 | -0.08 | 0.18 | 0.66 | -0.04 | 0.18 | 0.82 | -0.11 | 0.15 | 0.49 | -0.06 | 0.15 | 0.69 |
| 6 | 1.04 | 0.18 | <.00 | 1.08 | 0.18 | <.00 | 1.02 | 0.16 | <.00 | 1.07 | 0.16 | <.00 |
| Grade |  |  |  |  |  |  |  |  |  |  |  |  |
| 9 (ref) |  |  |  |  |  |  |  |  |  |  |  |  |
| 10 | 0.16 | 0.06 | 0.01 | 0.15 | 0.06 | 0.01 | 0.04 | 0.05 | 0.43 | 0.03 | 0.05 | 0.53 |
| 11 | 0.25 | 0.12 | 0.03 | 0.25 | 0.12 | 0.04 | 0.10 | 0.10 | 0.32 | 0.08 | 0.10 | 0.39 |
| 12 | 0.14 | 0.26 | 0.59 | 0.11 | 0.24 | 0.65 | 0.14 | 1.22 | 0.91 | 0.10 | 1.23 | 0.94 |
| Sex |  |  |  |  |  |  |  |  |  |  |  |  |
| Female (ref) |  |  |  |  |  |  |  |  |  |  |  |  |
| Male | -0.46 | 0.04 | <.00 | -0.47 | 0.04 | <.00 | -0.10 | 0.06 | 0.08 | -0.10 | 0.06 | 0.08 |
| Ethnicity |  |  |  |  |  |  |  |  |  |  |  |  |
| White (ref) |  |  |  |  |  |  |  |  |  |  |  |  |
| Asian | -0.10 | 0.09 | 0.25 | -0.10 | 0.09 | 0.26 | 0.20 | 0.12 | 0.08 | 0.20 | 0.12 | 0.08 |
| Other | -0.37 | 0.08 | <.00 | -0.36 | 0.08 | <.00 | -0.24 | 0.07 | 0.00 | -0.24 | 0.07 | 0.00 |
| Mixed | -0.13 | 0.08 | 0.11 | -0.12 | 0.08 | 0.13 | -0.07 | 0.08 | 0.37 | -0.06 | 0.08 | 0.42 |
| Spending Money |  |  |  |  |  |  |  |  |  |  |  |  |
| Zero (ref) |  |  |  |  |  |  |  |  |  |  |  |  |
| $1 to $5 | -0.12 | 0.07 | 0.09 | -0.12 | 0.07 | 0.10 | -0.10 | 0.07 | 0.15 | -0.09 | 0.07 | 0.17 |
| $6 to $10 | -0.08 | 0.07 | 0.25 | -0.08 | 0.07 | 0.25 | -0.08 | 0.07 | 0.19 | -0.08 | 0.06 | 0.20 |
| $11 to $20 | -0.05 | 0.07 | 0.45 | -0.05 | 0.07 | 0.45 | -0.05 | 0.05 | 0.31 | -0.05 | 0.05 | 0.34 |
| $21 to $40 | -0.05 | 0.07 | 0.49 | -0.04 | 0.07 | 0.54 | -0.11 | 0.06 | 0.06 | -0.11 | 0.06 | 0.08 |
| $41 to $100 | -0.07 | 0.07 | 0.30 | -0.07 | 0.07 | 0.34 | -0.06 | 0.07 | 0.35 | -0.05 | 0.07 | 0.45 |
| More than $100 | -0.16 | 0.10 | 0.09 | -0.15 | 0.10 | 0.13 | -0.16 | 0.09 | 0.08 | -0.13 | 0.09 | 0.13 |
| I don't know | -0.08 | 0.06 | 0.18 | -0.08 | 0.06 | 0.20 | 0.04 | 0.06 | 0.49 | 0.04 | 0.06 | 0.46 |
| Grades at Baseline | | | | | | | | | | | | |
|  | 0.84 | 0.03 | <.00 | 0.84 | 0.03 | <.00 | 0.58 | 0.03 | <.00 | 0.58 | 0.03 | <.00 |
| Average Daily MVPA | | | | | | | | | | | | |
|  |  |  |  | 0.00 | 0.00 | <.00 |  |  |  | 0.00 | 0.00 | <.00 |
| Meets Guidelines at Baseline | | | | | | | | | | | | |
| No (ref) |  |  |  |  |  |  |  |  |  |  |  |  |
| Yes | -0.17 | 0.04 | <.00 |  |  |  | -0.14 | 0.04 | 0.00 |  |  |  |
| Importance of Grades | | | | | | | | | | | | |
| Strongly Agree (ref) |  |  |  |  |  |  |  |  |  |  |  |  |
| Agree | -0.51 | 0.04 | <.00 | -0.51 | 0.04 | <.00 | -0.33 | 0.04 | <.00 | -0.33 | 0.04 | <.00 |
| Disagree/Strongly Disagree | -0.66 | 0.12 | <.00 | -0.67 | 0.12 | <.00 | -0.38 | 0.10 | 0.00 | -0.39 | 0.11 | 0.00 |
| Time Spent Sleeping (minutes/day) | | | | | | | | | | | | |
|  | 0.00 | 0.00 | 0.02 | 0.00 | 0.00 | 0.02 | 0.00 | 0.00 | 0.15 | 0.00 | 0.00 | 0.13 |
| Time Spent Doing Homework (minutes/day) | | | | | | | | | | | | |
|  | 0.00 | 0.00 | 0.00 | 0.00 | 0.00 | 0.00 | 0.00 | 0.00 | 0.01 | 0.00 | 0.00 | 0.01 |
| Intramural Sports Participation | | | | | | | | | | | | |
| No (ref) |  |  |  |  |  |  |  |  |  |  |  |  |
| Yes | 0.13 | 0.06 | 0.02 | 0.14 | 0.06 | 0.01 | 0.08 | 0.05 | 0.16 | 0.09 | 0.05 | 0.10 |
| Varsity Sports Participation | | | | | | | | | | | | |
| No (ref) |  |  |  |  |  |  |  |  |  |  |  |  |
| Yes | 0.14 | 0.05 | 0.01 | 0.15 | 0.05 | 0.01 | 0.20 | 0.04 | <.00 | 0.21 | 0.04 | <.00 |
| League Sports Participation | | | | | | | | | | | | |
| No (ref) |  |  |  |  |  |  |  |  |  |  |  |  |
| Yes | 0.03 | 0.05 | 0.55 | 0.05 | 0.05 | 0.32 | 0.00 | 0.05 | 0.92 | 0.02 | 0.05 | 0.67 |

Estimates generated using multinomial ordinal Generalized Estimating Equations (GEE) models.
